# *FNDC5* expression closely correlates with muscle fiber types in porcine *longissimus dorsi* muscle and regulates myosin heavy chains* (MyHCs)* mRNA expression in C2C12 cells

**DOI:** 10.7717/peerj.11065

**Published:** 2021-04-19

**Authors:** Xiao-Ming Men, Zi-Wei Xu, Xin Tao, Bo Deng, Ke-Ke Qi

**Affiliations:** Institute of Animal Husbandry and Veterinary Science, Zhejiang Academy of Agricultural Science, Hangzhou, Zhejiang, China

**Keywords:** FNDC5, Pig, Irisin, MyHC, C2C12

## Abstract

**Background:**

Irisin (a glycosylated protein) is cleaved from fibronectin type III domain-containing protein 5 (FNDC5), which is expressed mainly in animal muscle tissues and has multiple metabolic regulatory activities. However, their roles in controlling myofiber types in skeletal muscle remain unclear.

**Methodology:**

Two different commercial hybridized pigs, LJH (a crossed pig containing Chinese native pig genotypes) and DLY (Duroc × Landrace × Yorkshire) were selected to analyze *FNDC5* mRNA expression and the mRNA composition of four adult myosin heavy chain (*MyHC*) isoforms (IIIaIIxIIb) in the *longissimus dorsi* (LD) muscle. C2C12 myoblasts were cultured to investigate the effects of *FNDC5* on the four MyHCs mRNA expressive levels, using small interfering RNA for depletion and a eukaryotic expression vector carrying *FNDC5* for overexpression. ZLN005 (a small molecule activator of FNDC5’s upstream control gene *PGC1α*) or recombinant human irisin protein were also used.

**Results:**

In LD muscle, LJH pigs had the higher *FNDC5* mRNA level, and *MyHC I or IIa* proportion than DLY pigs (*P <  0.05*). For C2C12 cells in vitro, small interfering RNA (si-592) silencing of *FNDC5* expression markedly reduced *MyHC IIa* mRNA levels (*P <  0.05*), while *FNDC5* overexpression significantly increased* MyHC IIa* mRNA levels (*P <  0.05*). Exogenous irisin increased the mRNA levels of *PGC1α* (peroxisome proliferator-activated receptor gamma coactivator 1-alpha), *FNDC5*, *MyHCI*, *MyHCIIa*, *NRF1* (nuclear respiratory factor 1), *VEGF* (vascular endothelial growth factor), and* TFAM* (mitochondrial transcription factor A,) (*P <  0.05*), and the enzyme activities of SDH (succinate dehydrogenase), CK (creatine kinase), and MDH (malate dehydrogenase) in C2C12 myotubes (*P <  0.05*). These results showed that *FNDC5* mRNA expression had a significant association with the characteristics of myofiber types in porcine muscle, and participated in regulating *MyHCs* mRNA expression of C2C12 myogenic differentiation cells in vitro. *FNDC5* could be an important factor to control muscle fiber types, which provides a new direction to investigate pork quality via muscle fiber characteristics.

## Introduction

Muscle fiber is the basic unit of skeletal muscle in animals (Choi, Ryu & Kim, 2007). According to the contraction properties, mammalian skeletal muscle fibers are classified as slow-oxidative (type I), fast-oxidative (type IIa), intermediate (type IIx) and fast-glycolytic (type IIb) muscle fibers, and their myosin heavy chains (MyHCs) are mainly MyHC I, MyHCIIa, MyHC IIx and MyHC IIb isforms, respectively. Compared with glycolytic (type IIb) or intermediate (type IIx) muscle fibers, oxidative (type I and IIa) muscle fibers are richer in mitochondria and capillaries, have the stronger resistance to fatigue and rely more on oxidative phosphorylation to generate energy (Schiaffino & Reggiani, 2011). In contrast, glycolytic (type IIb) muscle fibers are prone to fatigue due to their rapid contraction (Schiaffino & Reggiani, 2011). Muscle fiber-types composition not only has a direct effects on the morphological characteristics and contractile function of skeletal muscle (Lee, Joo & Ryu, 2010; Ryu & Kim, 2005), but also affects meat water-holding capacity, tenderness, meat colour, pH and flavor in postmortem muscle through the different metabolic active factors, protein composition, fiber diameter and density among different muscle fiber-types. Thus, it has become a consensus that pork quality can be controlled through changing muscle fiber-types.

Irisin, as a glycosylated protein cleaved from fibronectin type III domain-containing protein 5 (FNDC5), was found to play certain roles in the processes of brown adipose cells and showed anti-obesity (Boström et al , 2012). Later, it was demonstrated that irisin could also improve multiple diseases, such as diabetes mellitus, chronic kidney disease, alcoholic fatty liver disease, metabolic syndrome, and neurological diseases (Cao et al., 2019). Irisin secretion or *FNDC5* gene expression can be regulated by exercise-activated and peroxisome proliferator-activated receptor gamma coactivator 1-alpha (*PGC1α* ) pathways (Boström et al , 2012; Cao et al., 2019). In the present study, the objective was to investigate the relationship between *FNDC5* gene expression and the mRNA composition of four adult MyHC isoforms(I, IIa, IIx and IIb) in porcine muscle, and the effects of FNDC5-irisin on the four *MyHCs* mRNA expression in C2C12 myoblast cells in vitro. The results could provide the new idea for us to control pork quality by regulating muscle-fiber types.

## Materials & Methods

### Animal feeding, muscle sampling, FNDC5 expression, and MyHC mRNA composition analysis

We selected 16 LJH (a crossed pigs containing Chinese native pig genotypes) and DLY (Duroc × Landrace × Yorkshire) weanling castrated pigs (eight pigs each hybrid combination), fed in Lvjia Yuan Livestock Industry Co., Ltd, Zhejiang Province, China. All animal feeding management and slaughtering processes were approved by the Laboratory Animal Management Committee of Zhejiang Academy of Agricultural Sciences in China (No. 2018110). At final body weights of 100–110 kg, these pigs were transferred to an abattoir by ordinary commercial truck (journey time ∼2.5 h) according to the commercial standard slaughtering process in China. The *longissimus dorsi* (LD) muscle located in the last 3–4 thoracic vertebrae was immediately collected after slaughter.

The total RNA in muscle was isolated using the Trizol method, and cDNA was synthesized using a ReverTra Ace qPCR RT Kit (Toyobo, Osaka, Japan). Quantitative real-time PCR (qPCR) was performed for the *FNDC5* mRNA level, with the *18s* rRNA gene as the reference gene. The *FNDC5* primer sequences were as follows: F-5′-tgcaggccatccattcag-3′, R-5′-ccacagagaccacga-3′, generating a 182 bp amplicon (Cai et al., 2017); *18s* primer sequences were as follows: F-5′-cccacggaatcgagaaagag-3′, R-5′-ttgacggaagggcacca-3′(Men et al., 2017).The relative quantification of *FNDC5* mRNA was calculated using the 2 ^−ΔΔCT^ method (Livak & Schmittgen, 2002).

According to the description in Men et al. (2017), the MyHC mRNA composition was analyzed using qPCR. The relative ratio of each MyHC-type mRNA was calculated as the corresponding copy number per mg of muscle sample divided by the sum of four MyHC-types mRNA, multiplied by 100.

### Culture, differentiation, and transfection of C2C12 myoblast cells

According to Zhou et al. (2013), C2C12 cells (an immortalized mouse myoblast cell line from the Shanghai Institute of Biochemistry and Cell Biology of Chinese Academy of Sciences, Shanghai, China) were cultured and induced to differentiate into myotubes in Dulbecco’s modified Eagle’s medium (DMEM) with 2% horse serum. Mycoplasma detection on C2C12 myoblast cells was carried out using a MycAway™ -Color One-Step Mycoplasma Detection Kit by YEASEN Biotech Co. Ltd., Shanghai city, China.

The small interfering RNA targeting *FNDC5* (si-592), the native oligonucleotide fragments (3′-uucuccgaacgugucacgutt-5′, 3′-acgugacacguucggagaatt-5′) for siRNA transfection, and the eukaryotic expression vector overexpressing *FNDC5* were supplied by GenePharma Biotechnology Co. Ltd., Shanghai city, China, according to the coding region sequence of *FNDC5* (NM_027402.4). The si-592 sequences included 3′-gccaguaugauaugaucaat-5′and 3′-uugaugaugauaucauacuggctt-5′and were transfected into C2C12 cells twice at day 0 and day 2 post inductive differentiation, respectively. The FNDC5 eukaryotic expression vector was constructed using plasmid pcDNA3.1(+), and transfected into C2C12 cells once at day 0 post inductive differentiation.

The transfection experiment was grouped as follows: nuclease-free water as the blank group, transfection reagent as the mock group, pcDNA3.1(+) plasmid the as vector-NC group, native oligonucleotide fragments as the si-NC group, si-592 fragment as FNDC5(-) group, and pcDNA3.1(+)-FNDC5 vector as FNDC5(+) group. According to the instructions of GP-RNA-mate kit supplied by GenePharma Biotechnology Co. Ltd, 0.4 mL of each transfection mixture was added into each well of a 6-well plate with 1.6 mL of fresh serum-free DMEM medium, and incubated at 37 °C for 5 h. After 4 days of incubation, the cultured cells were harvested for RNA or protein determination.

### ZLN005 or irisin treatment on C2C12 myoblasts

ZLN005, a small molecule activator of *PGC*-1*α*, was purchased from Cayman Chemical Company (Ann Arbor city, Michigan, USA). C2C12 cells (70–80% confluent) were induced in differentiation medium (DMEM+2% horse serum) containing various concentrations of ZLN005 (0, 5, and 10 µmol/mL) for 4 days and then collected. For the ZLN005-10 group, some cells were transfected with si-592, and harvested for RNA extraction. Recombinant human irisin protein was purchased from Pepro Tech Inc. Rocky Hill, NJ, USA. C2C12 cells were incubated in differentiation medium for 4 days, cultured in medium containing 0, 20, or 200 ng/mL irisin for 5 h, and then harvested for RNA extraction and metabolic enzyme activity assays.

### Quantitative real-time PCR (qPCR) and western blotting of cells

The RNA extraction, cDNA synthesis, and qPCR for C2C12 cells were performed similarly to the above descriptions for muscle tissue. All data were normalized against the mRNA expression of the glyceraldehyde-3-phosphate dehydrogenase (*GAPDH*) gene. Determinations were performed in triplicate. The primer sequences in Table 1 were designed and synthesized by GenePharma Biotechnology Co. Ltd, Shanghai city, China.

**Table 1:** List of genes and sequences of the primers for real-time quantitative PCR in vitro C2C12 cells.

Genes	Primer	Sequence 5′–3′	References
FNDC5	Forward Reverse	AGCTCAGAAGTAGAATGCGAGAG GGTGATAGGAGAAGATGGTGGTG	Chen et al. (2019)
GAPDH	Forward Reverse	AGACAGCCGCATCTTCTTGT CTTGCCGTGGGTAGAGTCAT	Chen et al. (2019)
MyHCI	Forward Reverse	TTGCTGTTATTGCCGCCATTG GAGTTGTCATTCCGAACTGTCTTG	Li et al. (2019)
MyHCIIx	Forward Reverse	CGAAGTTGCATCCCTAAAGGCAG CGAAAACGGCCATCTCGGC	Li et al. (2019)
MyHCIIb	Forward Reverse	GAAGGAGGGCATTGATTGGGAG TGTTCTTGAAGGAGGTGTCTGTCGC	Li et al. (2019)
MyHCIIa	Forward Reverse	TTCCAGAAGCCTAAGGTGGTC GCCAGCCAGTGATGTTGTAAT	Chen et al. (2018)
PGC1 *α*	Forward Reverse	CCAGTACAACAATGAGCCTGC CAATCCGTCTTCATCCACG	Chen et al. (2018)
NRF1	Forward Reverse	GCTGCTTTCAGTCCTTCTGG GTGTTCAGTTTGGGTCACTCC	Wadley, Choate & Mcconell (2007)
IL15	Forward Reverse	AATCCACCTTGACACATGGC AGGCTGGTTATCTGCTGACA	Waickman et al. (2017)
VEGF	Forward Reverse	CGTTTAACTCAAGCTGCCTCGC CTTCCAGGAGTACCCCGACGAGATA	Tang et al. (2010)
TFAM	Forward Reverse	CACCCAGATGCAAAACTTTCAG CTGCTCTTTATACTTGCTCACAG	Makiko et al. (2015)

Total protein extraction, SDS-PAGE gel electrophoresis, and western blotting for cells were performed according to Cai et al. (2017). The FNDC5 protein was separated using 12% SDS electrophoresis, transferred onto a cellulose acetate membrane, and then incubated with recombinant rabbit anti-FNDC5 monoclonal antibodies (ab174833, Abcam, Cambridge, UK), or a monoclonal anti- *β*-Actin antibody produced in mouse (A5441, Merck Life Science Co., Ltd, Shanghai city, China. Chemiluminesence detection was performed on an FR-1800 Luminescent and Fluorescent Biological Image Analysis System of Furi company, Shanghai city, China. The scanned images were processed and analyzed using Gel-Pro analyzer software from Media Cybernetics, Rockville, MD, USA. The relative content of FNDC5 protein in a sample was calculated through the ratio of the gray value to that of the internal reference actin.

### Detection of metabolic enzyme activity in cells

The harvested cell cultures were homogenized in one mL of phosphate buffered saline (pH 7.4), and then centrifuged at 1,000 rpm for 10 min at 4 °C. The supernatant was collected to detect the activities of succinate dehydrogenase (SDH), malate dehydrogenase (MDH), lactate dehydrogenase (LDH) and creatine kinase (CK), and protein concentration using the standard commercial kits from Nanjing Jiancheng Biochemical Institute, Nanjing city, China.. Enzyme activities were expressed as U/mg protein.

### Statistical analysis

All data are presented as the mean ±  standard deviation (SD) and all statistical analysis programs were operated in SPSS 16.0 (IBM Corp., Armonk, NY, USA). Data from porcine muscle tissue were analyzed using the *t*-test (*P* < 0.05) between LJH and DLY pig groups. Data from the cell experiments were analyzed using one-way analysis of variance and Duncan’s test (*P* < 0.05) among multiple treatment groups.

## Results

The variation of FNDC5 mRNA levels and MyHC mRNA composition in porcine LD muscle and their correlations

**Figure 1:**
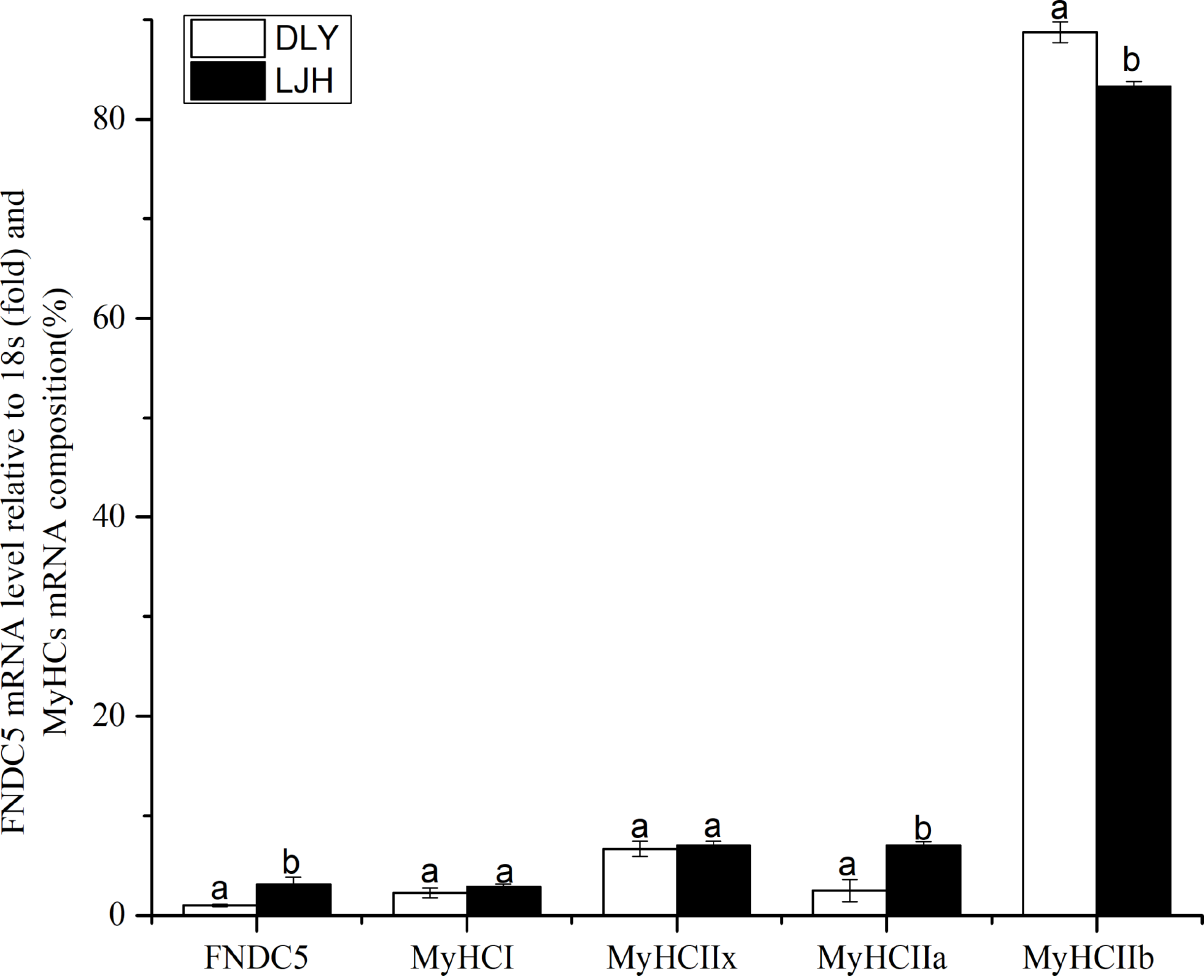
Difference of FNDC5 mRNA level and MyHCs mRNA composition in porcine LD muscle between LJH and DLY crossed pigs. Data are shown as means ± sd from eight pigs each crossed combination. The same letter on the label means *P* > 0.05; a different letter means *P* < 0.05. FNDC5, fibronectin type III domain-containing protein 5. LJH means a crossed pigs containing Chinese native pig genotypes, DLY means Duroc × Landrace × Yorkshire crossed pigs.

As shown in Fig. 1, LJH pigs had a higher *FNDC5* mRNA level and *MyHCI* or *IIa* mRNA proportions, and a lower *MyHCIIb* mRNA proportion in LD muscle than DLY pigs (*P* < 0.05).

### Knockdown or up expression of FNDC5 gene in C2C12 myoblast cells

**Figure 2:**
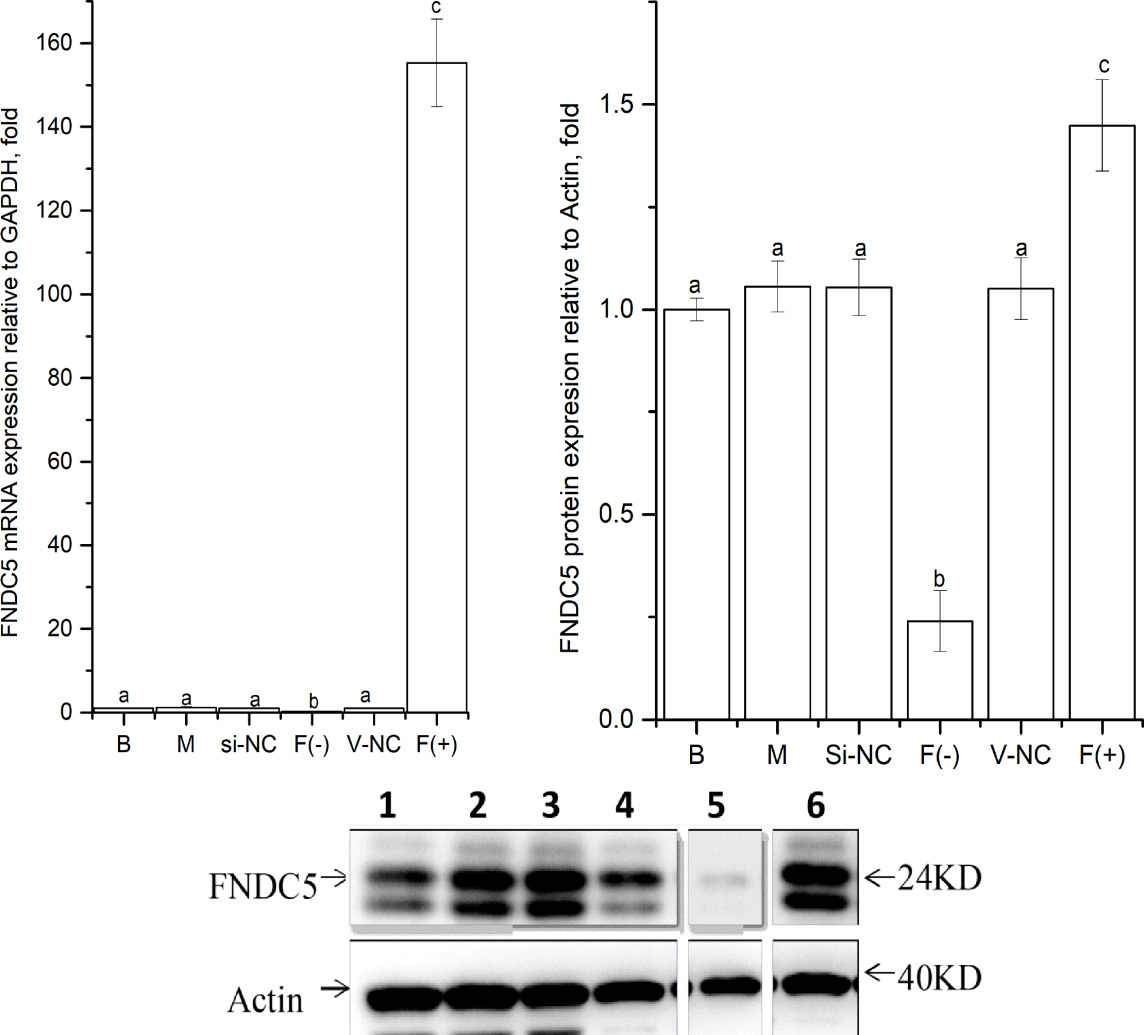
Effects of nucleic acid transfection on *FNDC5* mRNA (A) and protein (B) expressions in vitro in C2C12 myotubes. Each data point are shown as means ± sd from three repeated cell treatments. The same letter on the label means *P* > 0.05; a different letter means *P* < 0.05. In the images (C) of Western-Blot for FNDC5 protein expression, 1–6 means B, M, Si-NC, Vector-NC, si592/FNDC5(-),FNDC5(+).

As shown in Fig. 2, the expression levels of *FNDC5* mRNA and protein were not significantly different among the blank, mock, si-NC, and vector-NC groups (*P >  0.05*). In the FNDC5(-)group, the expression levels of *FNDC5* mRNA and protein were decreased significantly compared with those in the blank group by si-592 (*P <  0.05).* In the FNDC5(+) group, the expression levels of *FNDC5* mRNA and protein were increased significantly compared those in the blank group by pcDNA3.1(+)-FNDC5 vector (*P <  0.05).*

### Effects of down or up regulating FNDC5 expression on MyHCs mRNA levels in C2C12 myoblast cells

**Figure 3:**
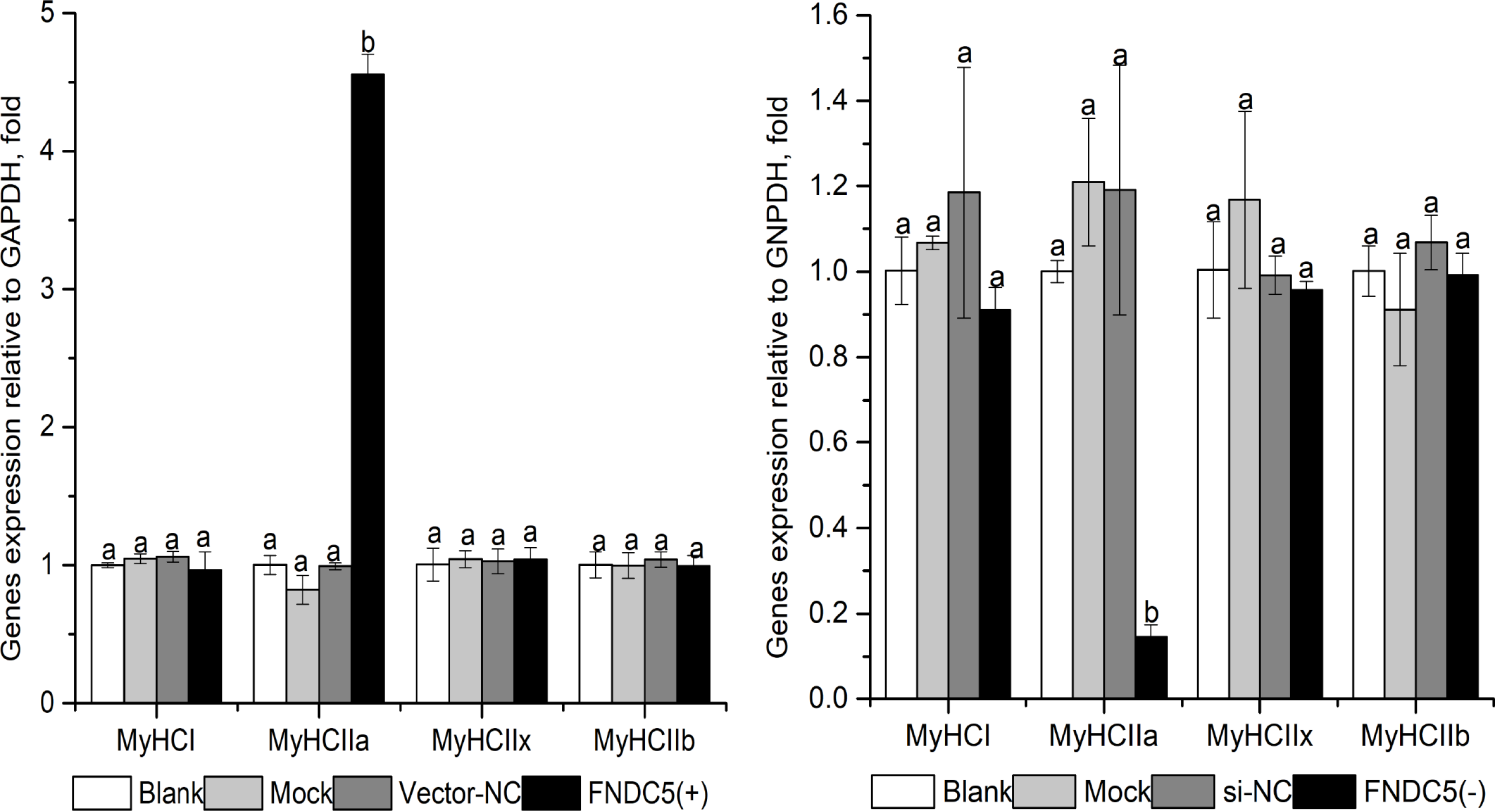
Effect of *FNDC5.* gene knockdown (A) or up-regulated (B) expression on *MyHCs* mRNA levels during C2C12 myogenic differentiation. Each data point are shown as means ± sd from three repeated cell treatments. The same letter on the label means *P* > 0.05; a different letter means *P* < 0.05. FNDC5, fibronectin type III domain-containing protein 5; MyHCs, myosin heavy-chains. *FNDC5 (-)* means *FNDC5* down-regulation by si592, *FNDC* (+) means *FNDC5* up-regulation by over-expression vector.

As shown in Fig. 3, *MyHCI*, *MyHCIIx* and *MyHCIIb* mRNA levels were not affected significantly in the *FNDC5* (-) or *FNDC5* (+)group (*P >  0.05*). *MyHCIIa* mRNA expression in the *FNDC5* (-) group were decreased significantly compared with that in other groups (*P <  0.05),* and increased significantly in the *FNDC5* (+) group compared that in other groups (*P <  0.05).*

### Effects of FNDC5 knockdown expression on PGC1*α*-FNDC5-MyHCs mRNA expression under ZLN005 in C2C12 myoblast cells

**Figure 4:**
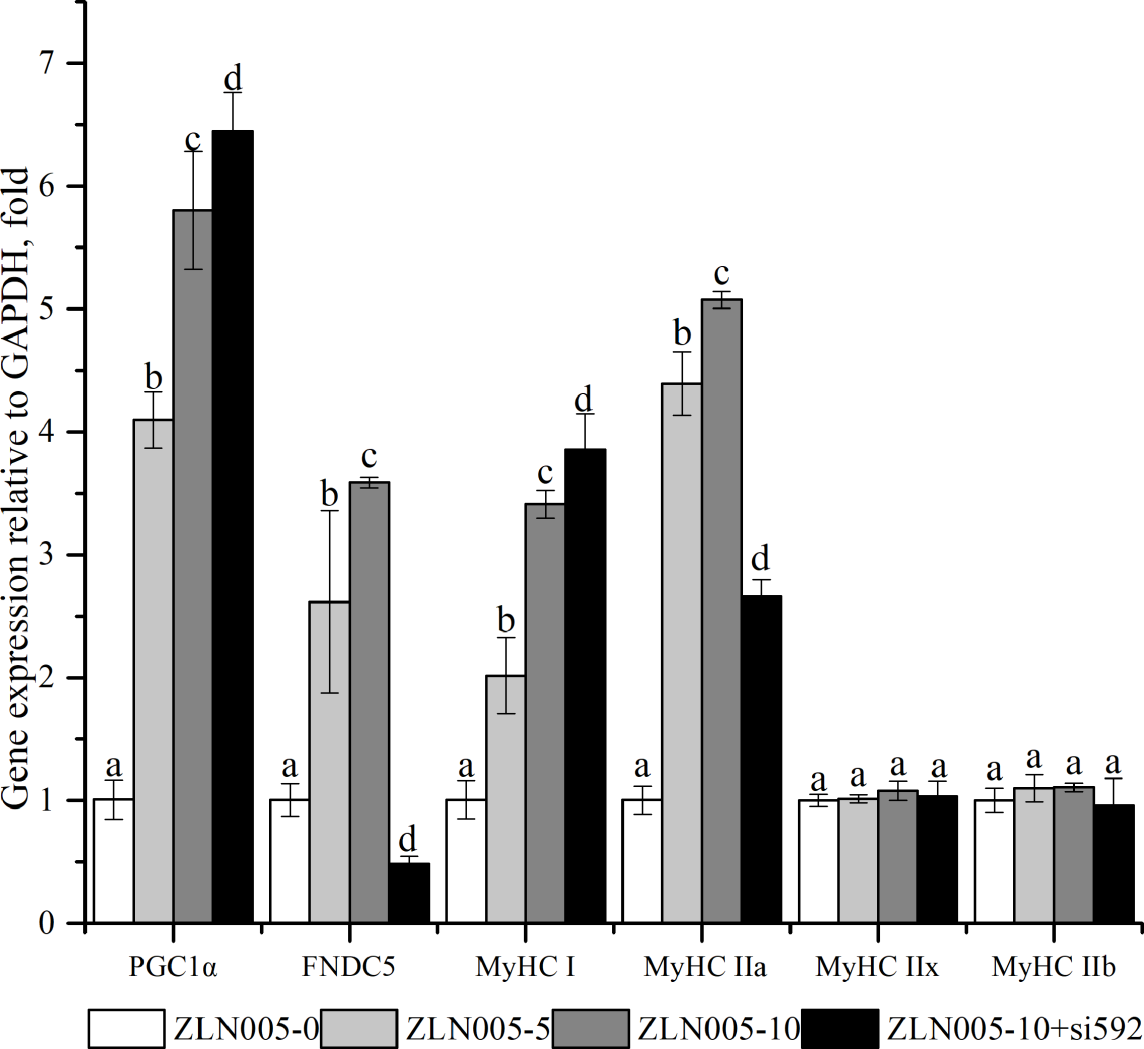
*PGC1α-FNDC5-MyHC* mRNA expressions in C2C12 myotubes with different amounts of ZLN005 and FNDC5(-) (si592) transfection. Each data point are shown as means ± sd from three repeated cell treatments. The same letter on the label means *P* > 0.05; a different letter means *P* < 0.05. FNDC5, fibronectin type III domain-containing protein 5; MyHC, myosin heavy-chain; PGC1 *α*, peroxisome proliferator-activated receptor gamma coactivator 1-alpha.

As shown in Fig. 4, compared with the ZLN005-0 group, the ZLN005-5 and ZLN005-10 groups showed significantly the increased expressive levels of *PGC1α*, *FNDC5*, *MyHCI* and *MyHCIIa* mRNA (*P* < 0.05). Compared with that in the ZLN005-10 group, ZLN005-10 + si592 group showed significantly the decreased expressive levels of *FNDC5* and *MyHCIIa* mRNA (*P* <  0.05).

### Effects of adding irisin on PGC1*α*-FNDC5-MyHCs mRNA expression in C2C12 myoblast cells

**Figure 5:**
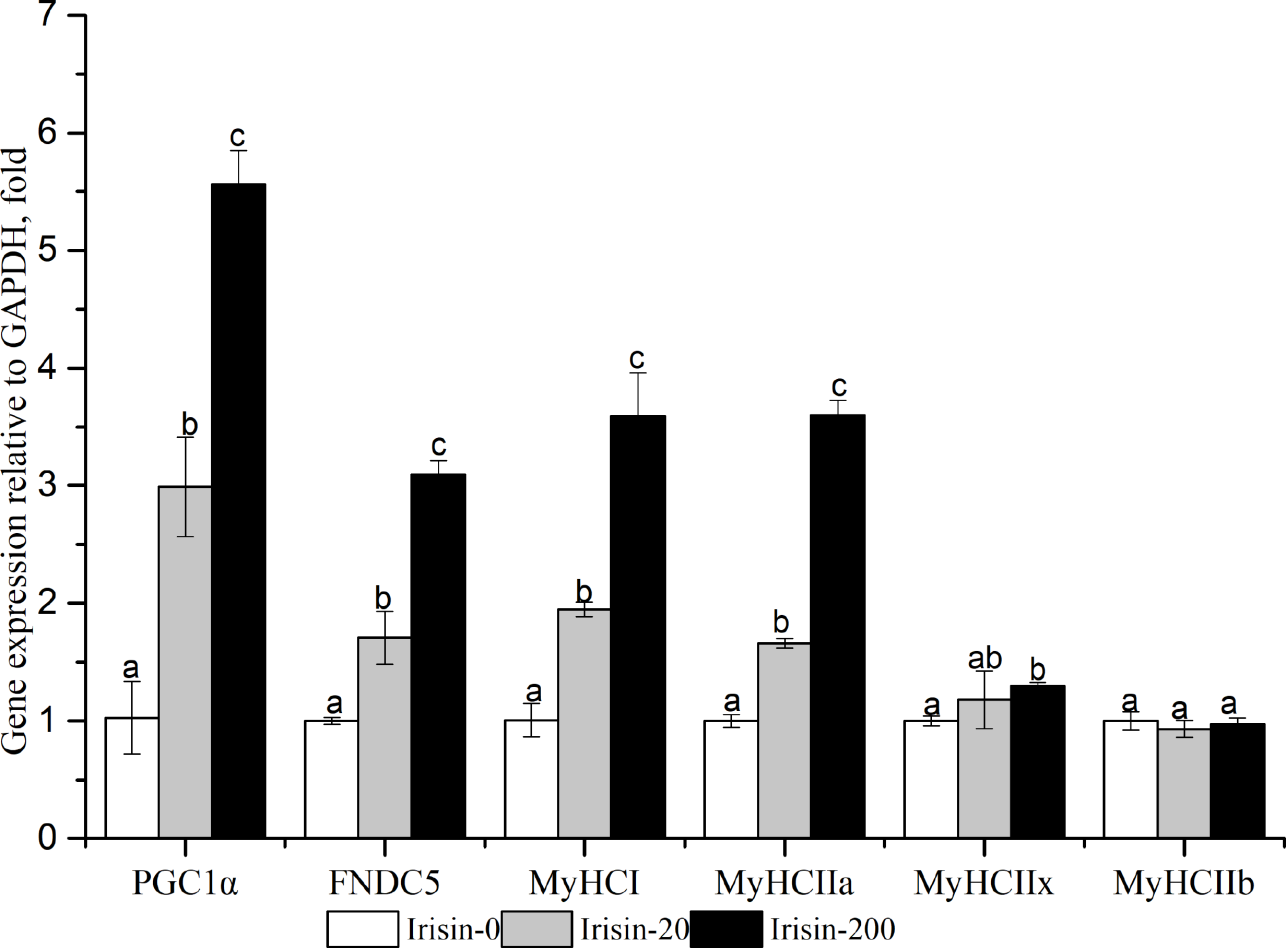
Effects of adding irisin on *PGC1α*, *FNDC5* and *MyHC* genes expressions in C2C12 myotubes with different amounts of irisin. Each data point are shown as means ± sd from three repeated cell treatments. The same letter on the label means *P* > 0.05; a different letter means *P* < 0.05. FNDC5, fibronectin type III domain-containing protein 5; MyHCs, myosin heavy-chain; PGC1 *α*, peroxisome proliferator-activated receptor gamma coactivator 1-alpha.

As shown in Fig. 5, *PGC1α* , *FNDC5*, *MyHCI*, and *MyHCIIa* mRNA expressions were significantly upregulated with the increasing irisin addition (*P <  0.05*).The Irisin-200 group also showed significantly increased *MyHCIIx* mRNA expression compared with that in the irisin-0 group (*P* < 0.05).

### Effects of adding irisin on metabolic enzyme activities and other downstream gene expression levels in C2C12 myoblast cells

**Figure 6:**
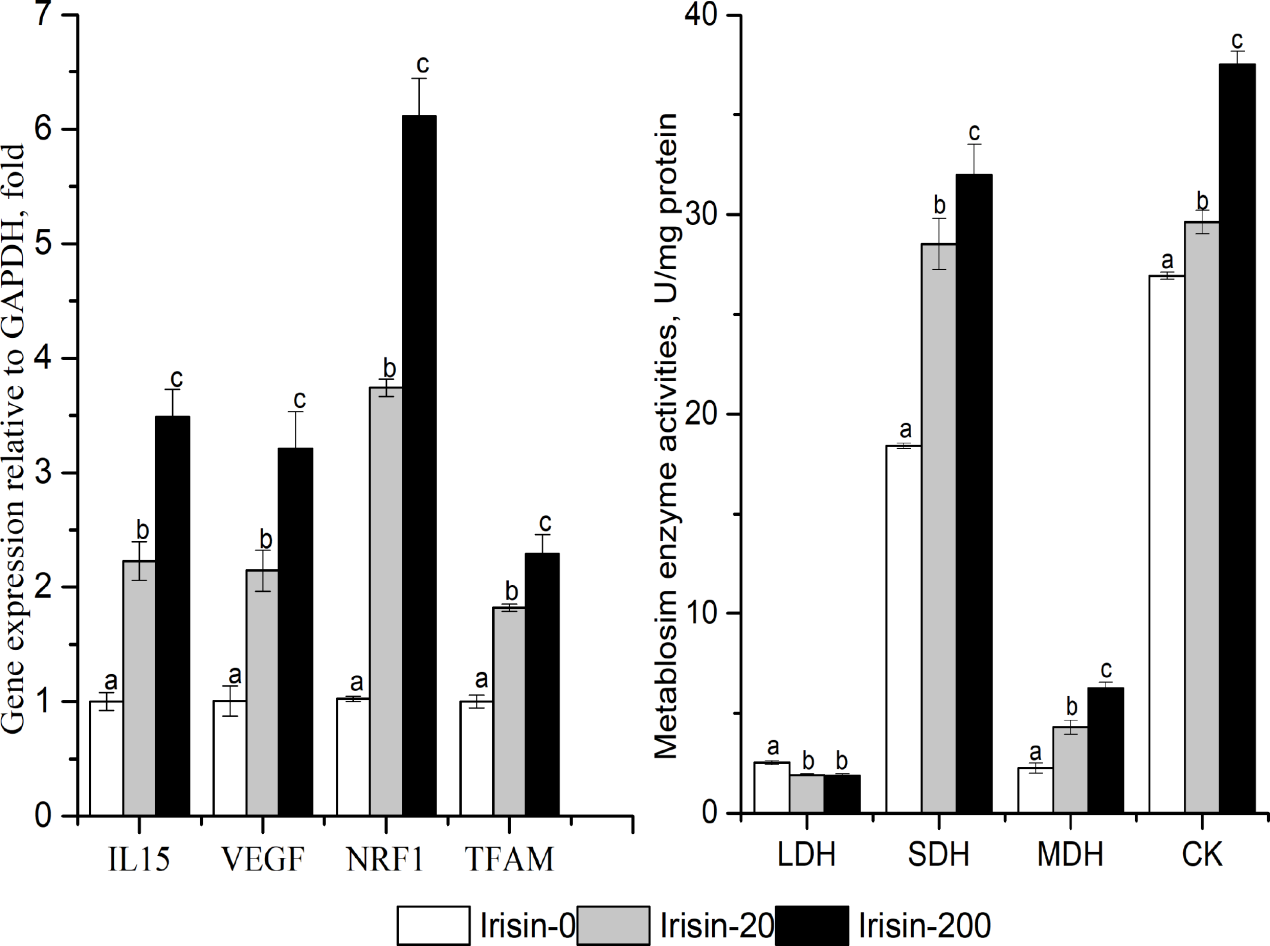
Effects of adding irisin the metabolic enzyme activities(B) and other downstream genes expressions (A) in C2C12 myotubes. Each data point are shown as means ± sd from three repeated cell treatments. The same letter on the label means *P* > 0.05; a different letter means *P* < 0.05. CK, creatine kinase; SDH, Succinate dehydrogenase; MDH, Malate dehydrogenase; LDH, lactate dehydrogenase; *IL15,* interleukin 15; *VEGF,* vascular endothelial growth factor; *NRF1,* nuclear respiratory factor 1; *TFAM,* transcription factor A mitochondrial.

As shown in Fig. 6, the enzyme activities of SDH, MDH and CK, and the mRNA expression levels of *IL15* (interleukin 15), *VEGF* (vascular endothelial growth factor), *NRF1* (nuclear respiratory factor 1), and *TFAM* (transcription factor A, mitochondrial) increased with increasing irisin addition in C2C12 myotubes (*P* < 0.05). The activity of LDH was significantly lower in the irisin-20 and irisin-200 groups than that in the Irisin-0 group (*P* < 0.05).

## Discussion

To date, various cellular signaling pathway factors mediating muscle fiber-types conversion have been identified, such as the Ca ^2+^/calcineurin, NFAT, AMP-activated protein kinase (AMPK), peroxisome proliferator-activated receptor gamma coactivator 1-alpha (PGC1*α*), mitogen-activated protein kinase, myocyte enhancer factor 2 (MEF2), Wnts, forkhead transcriptional factor (FoxO1) and myofiber regulation factors (MRFs) (Li et al., 2019; Rockl et al., 2007; Handschin et al., 2007a; Handschin et al., 2007b; Scharf et al., 2013; Anderson et al., 2015; Cisternas et al., 2014; Yuan et al., 2011; Li et al., 2014).

As a key factor in regulating skeletal muscle fiber type transformation (especially oxidative fiber formation), *PGC1α* can induced *FNDC5* expression and irisin secretion in skeletal muscle (Boström et al , 2012; Svensson & Handschin, 2014; Bai et al., 2019). *PGC1α*, *FNDC5* and oxidative-type muscle fiber can be induced some the same factors, such as endurance training, stretching, mechanical load, outdoor exercise and cold environment (Men, Tao & Xu, 2016). In addition, the increasing mitochondrion is an important marker of oxidative-type muscle fibers formation, also the significant result of *FNDC5* or irisin action. *FNDC5* also plays a regulatory role in fatty acid metabolism and glucose utilization (Guo et al., 2019; Cao et al., 2019), and interacts with myogenic regulatory factors (Bai et al., 2019). Ellefsen et al. (2014) found that *FNDC5* gene expression was correlated with proportions of aerobic muscle fibers in untrained women, but the relationship disappeared in trained ones. These existed researches suggested a closely association between FNDC5 and muscle fiber-types. The study found that FNDC5 mRNA expression and MyHC I and IIa mRNA percentage exhibited the same direction difference in LD muscle from different genotype pigs, which confirmed the closely association between *FNDC5* gene and muscle fiber types.

To further explore the underlying mechanism of FNDC5-irisin effects on muscle fiber types, the skeletal muscle model cells C2C12 were used. We found that *MyHCIIa* mRNA and *FNDC5* mRNA or protein expression levels were increased by transfection of the FNDC5-vector during C2C12 myogenic differentiation in vitro. By contrast, *MyHCIIa* mRNA, and *FNDC5* mRNA or protein expression levels were decreased after transfection with si-FNDC5 (si-592). These results suggested that *FNDC5* might be involved in the formation of fast-oxidative muscle fibers during the myogenic differentiation of C2C12 cells in vitro.

In previous reports, *PGC1α* was identified as the upstream dependent factor controlling *FNDC5* expression and irisin secretion (Boström et al , 2012). For instance, the *PGC1α*-*FNDC5*-*UCP1* axis functions in regulating metabolism in adipose tissue or cells (Sanchez-Delgado et al., 2015). In the present study, the addition of ZLN005 increased the mRNA levels of *PGC1α*, *FNDC5*, *MyHCI*, and *MyHCIIa* in C2C12 *myoblast* cells. This phenomenon accorded with the transcriptional activation by ZLN005 on *PGC1α* (Zhang et al., 2013), and the promoting effects of *PGC1α* on oxidative-type myofiber formation were verified in mouse skeletal muscle (Handschin, 2009) and porcine skeletal muscle (Lin, Hangschin & Spiegelman, 2005; Ying et al., 2016). Therefore, the *PGC1α*-*FNDC5*-*MyHCs* pathway of C2C12 cells was activated by ZLN005.

To further reveal the effects of *FNDC5* gene on *PGC1α*-regulated muscle fiber-types, we knocked down *FNDC5* gene expression in the context of ZLN005-mediated activation of the *PGC1α* signaling pathway. The result showed that *FNDC5* knockdown expression significantly suppressed *MyHCIIa* mRNA expression under *PGC1α* activation, but had no effects on *MyHCI*, *MyHCIIx* or *MyHCIIb* mRNA levels. FNDC5 might be directly involved in PGC1*α*-induced *MyHCIIa* expression rather than *MyHCI* expression in C2C12 *myoblast* cells.According to the previous reports, irisin has a wide range of biological functions on various tissues or organs with the help of circulation system (Polyzos et al., 2013; Hawley et al., 2014; Cao et al., 2019). Our results confirmed that irisin could activate the upstream PGC1*α*-FNDC5 pathway in C2C12 cells. According to Ye et al. (2019), irisin could promote glucose uptake in C2C12 cells by activating the AMP-activated protein kinase (*AMPK*) signaling pathway (Ye et al., 2019). At the same time, the *AMPK* pathway can activate mitochondrial biogenesis through activating *PGC1α* (Liang et al., 2018). These observations indicated that irisin functions via an autocrine regulatory mechanism, which would provide a theoretical explanation for irisin’s effects on the differentiation of muscle fiber-types, mitochondrial synthesis, and tissue metabolism.

Our results supported the functions of *PGC1α* gene. First, the activities of the oxidative metabolic enzymes (SDH and MDH) and the expression of oxidative muscle-fiber genes (*MyHC* I and *MyHC* IIa) were increased by irisin treatment in C2C12 myotubes. Second, irisin treatment increased the expressions of *IL15*, *NRF1*, *VEGF*, and *TFAM* in a dose-dependent manner. *FNDC5*, *IL15*, *NRF1*, *VEGF*, and *TFAM* are important downstream target genes of PGC1*α* (Cao et al., 2019; Jessica et al., 2009; Chen et al., 2018; Chen et al., 2019). In muscle cells, *IL15* can stimulate glucose transport and oxidation (Scharf et al., 2013), *NRF1* and *TFAM* participate in mitochondrial synthesis (Yu et al., 2014; Makiko et al., 2015), and *VEGF* can stimulate angiogenesis and is associated with the formation of oxidative muscle-fibers (Li, 2014).

## Conclusions

This study investigate the intermediate effects of *FNDC5* gene on muscle fiber types. In porcine muscle, the differences between two different porcine populations was uniform in *FNDC5* mRNA level, the mRNA proportions of *MyHC I, MyHCIIa, or MyHCIIx,* but contrasted in *MyHCIIb* mRNA proportion.. In C2C12 myoblast cells in vitro, *FNDC5* gene was demonstrated to be directly involved in *MyHCIIa* mRNA expression.Irisin could activate *PGC1α* gene expression, a upstream dependent gene of *FNDC5*, and further play a more extensive role in skeletal muscle cells, including the autocrine regulation on *FNDC5* expression, mitochondrial functions, energy metabolic enzyme activities. *FNDC5* gene could be an important factor to control muscle fiber types, which would provide the newdirection to investigate pork quality by muscle fiber characteristics.

##  Supplemental Information

10.7717/peerj.11065/supp-1 Supplemental Information 1 Raw data of FNDC5mRNA expression and MyHCs mRNA composition in porcine muscle Each data point indicates the value from one pig.

10.7717/peerj.11065/supp-2 Supplemental Information 2 Raw data of genes expression or enzymes activity of C2C12 cells in vitro Each data point indicates the average performance of three repeats measures of cells

10.7717/peerj.11065/supp-3 Supplemental Information 3 Original picture of WB

10.7717/peerj.11065/supp-4 Supplemental Information 4 Explanation regarding the double band seen in the Western blot and its the antibody blocking test

10.7717/peerj.11065/supp-5 Supplemental Information 5 Mycoplasma detection The instructions of MycAway™ -Color One-Step Mycoplasma Detection Kit and detection results.

## Additional Information and Declarations

### Data Availability

The following information was supplied regarding data availability: Raw data showing MyHCs mRNA composition in porcine muscle and various genes expressions in C2C12 cells are available in the Supplementary Files.

## References

[ref-1] Anderson CM, Hu J, Barnes RM, Heidt AB, Cornelissen I, Black BL (2015). Myocyte enhancer factor 2C function in skeletal muscle is required for normal growth and glucose metabolism in mice. Skeletal muscle.

[ref-2] Bai Y, Bi H, Li L, Li J, Yu X, Ren H, Li Y, Ji Y, Hi Li, Wang H (2019). Effects of myostatin deficiency on PGC-1*α* and FNDC5 expression in three different murine muscle types. Acta Histochemica.

[ref-3] Boström P, Wu J, Jedrychowski MP, Korde A, Ye L, Lo JC, Rasbach KA, Boström EA, Choi JH, Long JZ, Kajimura S, Zingaretti MC, Vind BF, Tu H, Cinti S, Højlund K, Gygi SP, Spiegelman BM (2012). A PGC1- *α*-dependent myokine that drives brown-fat-like development of white fat and thermogenesis. Nature.

[ref-4] Cai CB, Xiao GJ, Qian LL, Jiang SW, Li B, Xie SW, Gao T, An XR, Cui WT, Li K (2017). Gene location, expression, and function of FNDC5 in Meishan pigs. Scientific Reports.

[ref-5] Cao RY, Zheng HC, Damian R, Yang J (2019). FNDC5: a novel player in metabolism and metabolic syndrome. Biochimie.

[ref-6] Chen SQ, Ding LN, Zeng NX, Liu HM, Zheng SH, Xu JW, Li RM (2019). Icariin induces irisin/FNDC5 expression in C2C12 cells via the AMPK pathway. Biomedicine and Pharmacotherapy.

[ref-7] Chen ZB, Tao SW, Li XH, Yao QH (2018). Resistin destroys mitochondrial biogenesis by inhibiting the PGC-1*α*/NRF1/TFAM signaling pathway. Biochemical and Biophysical Research Communications.

[ref-8] Choi YM, Ryu YC, Kim BC (2007). Influence of myosin heavy and light chain iso-forms on early postmortem glycolytic rate and pork quality. Meat Science.

[ref-9] Cisternas P, Henriquez JP, Brandan E, Inestrosa NC (2014). Wnt signaling in skeletal muscle dynamics: myogenesis, neuromuscul, synap, fibros. Molecular neurobiology.

[ref-10] Ellefsen S, Vikmoen O, Slettalkken G, Whist JE, Nygaard H, Hollan I, Rauk I, Vegge G, Strand TA, Raastad T, Rannestad BR (2014). Irisin and FNDC5: effects of 12-week strength training, and relations to muscle phenotype and body mass composition in untrained women. European Journal of Apply Physiology.

[ref-11] Guo Q, Wei X, Hu H, Yang DQ, Zhang B, Fan X, Liu J, He H, Oh Y, Wu Q, Zhang Y, Wang C, Liu C, Gu N (2019). The saturated fatty acid palmitate induces insulin resistance through Smad3-mediated down-regulation of *FNDC5* in myotubes. Biochemical and Biophysical Research Communications.

[ref-12] Handschin C (2009). The biology of PGC-1a and its therapeutic potential. Cell.

[ref-13] Handschin C, Chin S, Li P, Liu F, Maratos-Flier E, Lebrasseur NK, Yan Z, Spiegelma BM (2007a). Skeletal muscle fiber-type switching, exercise intolerance, and myopathy in PGC-1alpha muscle-specific knock-out animals. Journal of Biological Chemistry.

[ref-14] Handschin C, Chin S, Li P, Liu F, Maratos-Flier E, Lebrasseur NK, Yan Z, Spiegelman BM (2007b). Skeletal muscle fiber-type switching, exercise in tolerance, and myopathy in PGC-1*α* muscle-specific knock-out animals. Journal of Biological Chemistry.

[ref-15] Hawley JA, Hargreaves M, Joyner MJ, Zierath JR (2014). Integrative Biology of Exercise. Cell.

[ref-16] Jessica C, Jorge R, Gupta RK, Thom R, Shoag J, Rowe GC, Sawada N, Raghuram S, Arany Z (2009). The transcriptional coactivator PGC-1alpha mediates exercise-induced angiogenesis in skeletal muscle. Proceedings of the National Academy of Sciences of the United States of America.

[ref-17] Lee SH, Joo ST, Ryu YC (2010). Skeletal muscle fiber type and myofibrillar proteins in relation to meat quality. Meat Science.

[ref-18] Li BJ, Li PH, Wu WJ, Li QF, Huang RH, Liu HL (2014). Progresses in research of the mechanisms of skeletal muscle fiber formation. Scientia Agricultura Sinica.

[ref-19] Li LX, Wang J, Bai Y, Li JW, Yu XJ, Luo XM, Zhu ZW, He XY, Dong YJ, Li HQ, Wang HD (2019). Effect of hypoxia on the muscle fiber switching signal pathways CnA/NFATc1 and myostatin in mouse myocytes. Acta Histochemica.

[ref-20] Li Y (2014). The effect of VEGF activating transcription factor in therapeutic angiogenesis and skeletal muscle fiber in the mouse with hindlimb ischemia. Journal of Vascular Surgery.

[ref-21] Liang TY, Wu JP, Liu T, Bai Y, Zhang R (2018). Recent progress in classification and transformation mechanism of muscle fiber types. Meat Research.

[ref-22] Lin J, Hangschin C, Spiegelman BM (2005). Metabolic control through the PGC-1 family of transcription coactivators. Cell Metabolism.

[ref-23] Makiko Y, Ayano N, Yuri S, Ayako S, Mariko T, Sakuka T, Kazuo K, Kaoruko I (2015). Dietary isoflavone daidzein promotes Tfam expression that increases mitochondrial biogenesis in C2C12 muscle cells. The Journal of Nutritional Biochemistry.

[ref-24] Men XM, Deng B, Tao X, Qi KK, Xu ZW (2017). Wnt gene expression in adult porcine longissimus dorsi and its association with muscle fiber type, energy metabolism, and meat quality. Journal of Integrative Agriculture.

[ref-25] Men XM, Tao X, Xu ZW (2016). Irisin-processor gene: molecular structure, expressive regulation, biological functions and the associations with skeletal muscle fiber types. Chinese Journal of Animal Nutrition.

[ref-26] Polyzos SA, Kountouras J, Shields K, Mantzoros CS (2013). Irisin: a renaissance in metabolism?. Metabolism: Clinical and Experimental.

[ref-27] Rockl KSC, Hirshman MF, Brandauer J, Fujii N, Witters LA, Goodyear LJ (2007). Skeletal muscle adaptation to exercise training: AMP-activated protein kinase mediates muscle fiber type shift. Diabetes.

[ref-28] Ryu YC, Kim BC (2005). The relationship between muscle fiber characteristics, postmortem metabolic rate, and meat quality of pig longissimus-dorsi muscle. Meat Science.

[ref-29] Sanchez-Delgado G, Martinez-Tellez B, Olza J, Aguilera CM, Á Gil, Ruiz JR (2015). Role of exercise in the activation of brown adipose tissue. Annals of Nutrition and Metabolism.

[ref-30] Scharf M, Neef S, Freund R, Geers-Knorr C, Franz-Wachtel M, Brandis A, Krone D, Schneider H, Groos S, Menon MB, Chang KC, Kraft T, Meissner JD, Boheler KR, Maier LS, Gaestel M, Scheibe RJ (2013). Mitogen-activated protein kinase-activated protein kinases 2 and 3 regulate SERCA2a expression and fiber type composition to modulate skeletal muscle and cardiomyocyte function. Molecular and Cellular Biology.

[ref-31] Schiaffino S, Reggiani C (2011). Fiber types in mammalian skeletal muscles. Physiological Reviews.

[ref-32] Svensson K, Handschin C (2014). Modulation of PGC-1a activity as a treatment for metabolic and muscle-related diseases. Drug Discovery Today.

[ref-33] Tang K, Feng CX, Wagner PD, Breen EC (2010). Exercise-induced VEGF transcriptional activation in brain, lung and skeletal muscle. Respiratory Physiology & Neurobiology.

[ref-34] Wadley GD, Choate J, Mcconell GK (2007). NOS isoform-specific regulation of basal but not exercise-induced mitochondrial biogenesis in mouse skeletal muscle. Journal of Physiology.

[ref-35] Waickman AT, Ligons DL, Hwang SJ, Park JY, Lazarevic V, Sato N, Hong C, Park JH (2017). CD4 effector T cell differentiation is controlled by IL-15 that is expressed and presented in trans. Cytokine.

[ref-36] Ye X, Shen Y, Ni C, Ye J, Xin Y, Zhang W, Ren Y (2019). Irisin reverses insulin resistance in C2C12 cells via the p38-MAPK-PGC-1*α* pathway. Peptides.

[ref-37] Ying F, Zhang L, Bu G, Xiong YZ, Bo Zuo (2016). Muscle fiber-type conversion in the transgenic pigs with overexpression of PGC1*α* gene in muscle. Biochemical and Biophysical Research Communications.

[ref-38] Yu J, Xiao Y, Liu J, Ji Y, Liu H, Xu J, Jin X, Liu L, Guan MX, Jiang P (2014). Loss of MED1 triggers mitochondrial biogenesis in C2C12 cells. Mitochondrion.

[ref-39] Yuan Y, Shi XE, Liu YG, Yang GS (2011). FoxO1 regulates muscle fiber-type specification and inhibits calcineurin signaling during C2C12 myoblast differentiation. Molecular and Cellular Biochemistry.

[ref-40] Zhang LN, Zhou HY, Fu YY, Li YY, Wu F, Gu M, Wu LY, Xia CM, Dong TC, Li JY, Shen JK, Li J (2013). Novel small-molecule PGC-1alpha transcriptional regulator with beneficial effects on diabetic db/db mice. Diabetes.

[ref-41] Zhou LN, Wang Y, Li QY, Xu HJ, Lan XF, Zhang XJ (2013). Culture and differentiation of C2C12 cells for identification of skeletal muscular fibers. Journal of Shanghai Jiaotong University(Medical Science).

