# Supplementary figures and images for: FNDC5 expression closely correlates with muscle fiber types in porcine longissimus dorsi muscle and regulates myosin heavy chains (MyHCs) mRNA expression in C2C12 cells
Source: PeerJ. 2021 Apr 19;9:e11065. doi: 10.7717/peerj.11065 (PMC8061570; doi:10.7717/peerj.11065)

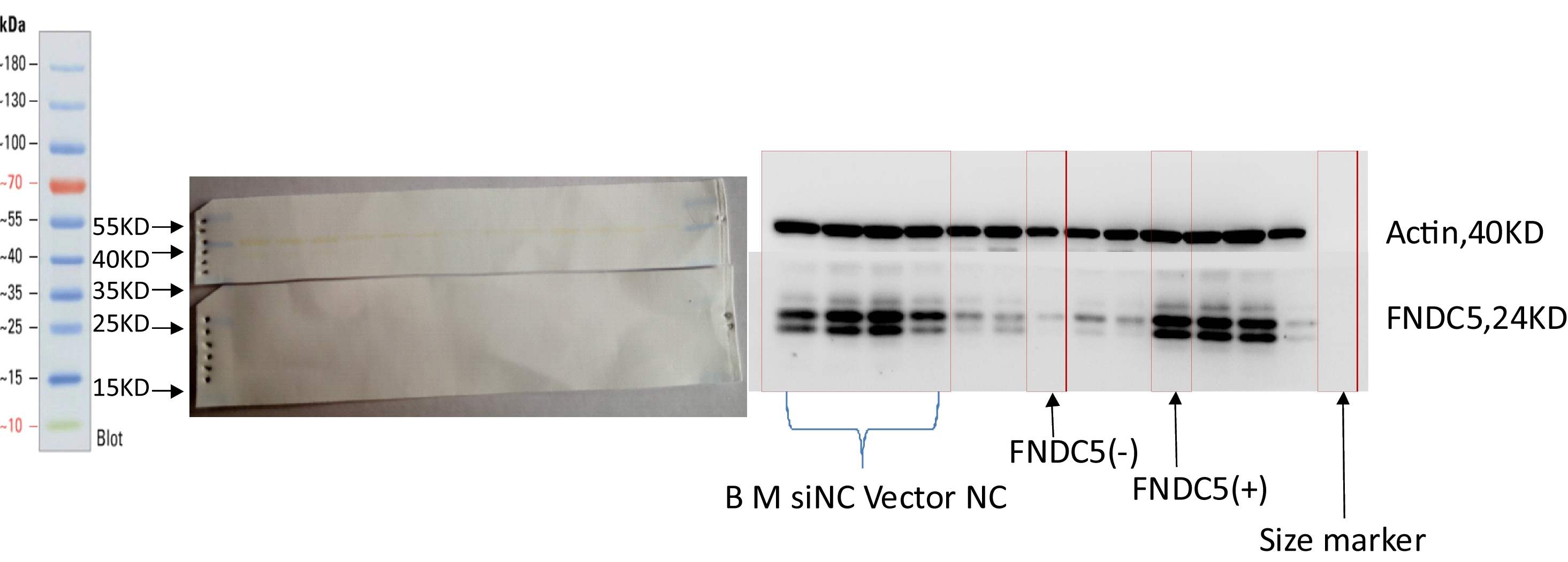

Supplement: Supplemental Information 3 [file peerj-09-11065-s003.jpg]
