# Supplementary material for: FNDC5 expression closely correlates with muscle fiber types in porcine longissimus dorsi muscle and regulates myosin heavy chains (MyHCs) mRNA expression in C2C12 cells
Source: PeerJ. 2021 Apr 19;9:e11065. doi: 10.7717/peerj.11065 (PMC8061570; doi:10.7717/peerj.11065)
Supplement: Supplemental Information 4 [file peerj-09-11065-s004.pdf]

我购买了**ab174833**，单克隆抗体。在目的蛋白附近有一个接近的条带。想咨询一下，具体原因和可行的解决方案。谢谢 **[事件: 201220-000066]**
发件人: "Abcam Scientific Support" <cn.technical@abcam.com>
收件人: menxiaoming@126.com
抄 送:
发送时间: 2020-12-21 13:55

abcam

咨询编号 201220-000066

创建日期: Sunday, Dec 20, 2020 - 16:42
上次更新日期: Monday, Dec 21, 2020 - 13:55

主题 我购买了ab174833，单克隆抗体。在目的蛋白附近有一个接近的条带。想咨询一下，具体原因和可行的解决方案。谢谢

由E-mail来自我们的回复 Monday, Dec 21, 2020 - 13:55

老师您好，

对于您使用ab174833于实验里没有获得理想结果, 我们感到非常抱歉。

根据UniProtKB - Q8NAU1 (FNDC5_HUMAN)（https://www.uniprot.org/uniprot/Q8NAU1#ptm_processing） 显示该蛋白存在20 kDa的isoform 2和剪切掉长为31个氨基酸的信号肽的片段，两者的大小都约为20 kDa均可能导致其在WB中显现出双带。isoform 2的长度为1-181 共181个氨基酸，剪切掉信号肽片段的长度为32-212共181个氨基酸。若要进行实验验证需要选择其他免疫原的抗体，验证isoform 2 时需要使用免疫原在182-212之间的抗体，验证信号肽时需选择免疫原在1-31之间的抗体；此外若有文献资料做与您相同的样本，其结果对您的分析也有帮助。

我们查找到一篇文献在进行WB实验时也检测到多条带，您可查看其详细内容。PMID:29176631

希望以上回复能够帮助到您，您有任何问题可随时联系我们。

Chunye Chen
Scientific Support Specialist
www.abcam.cn
+86 21 20700500

We are committed to continually improving our service and value your input. You may receive a 'HaveYourSay' survey email. Please share your thoughts.

由E-mail来自您的咨询 Sunday, Dec 20, 2020 - 16:43

您发给我的信件已经收到。

网易126邮箱--专业电子邮局，2280兆超大空间，支持超大附件，全国唯一24小时客户服务。
欢迎您来注册使用。网易126邮箱：http://www.126.com

This is an automatic reply, confirming that your e-mail was received.Thank you

由自动答复 Sunday, Dec 20, 2020 - 16:42

感谢您联系Abcam! 我们已经收到您的咨询邮件，在多数情况下，您可望尽快得到答复。

您的查询编号为:201220-000066。如果您需要就此事再次联系我们，请参考这个参考号码，这将帮助我们查找到您最初的咨询。

为方便查询，您可在 http://www.abcam.com/account/inquiry 登入您的帐户，点击“查询”，便可看到过去六个月内您得到的所有回复。

我们期待为您服务!

由E-mail来自您的咨询 Sunday, Dec 20, 2020 - 16:42

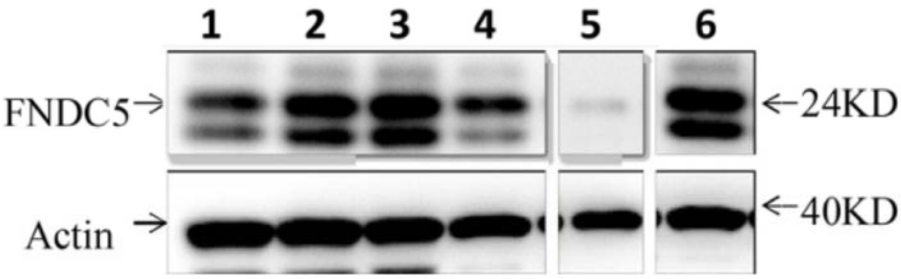

2020-12-20

nenxiaoming

我购买了ab174833，单克隆抗体。在目的蛋白附近有一个接近的条带。想咨询一下，具体原因和可行的解决方案。谢谢 [事件: 201220-000066]
发件人: "Abcam Scientific Support" <cn.technical@abcam.com>
收件人: menxiaoming@126.com
抄 送:
发送时间: 2020-12-23 13:28

咨询编号 201220-000066

创建日期: Sunday, Dec 20, 2020 - 16:42
上次更新日期: Wednesday, Dec 23, 2020 - 13:28
主题 我购买了ab174833，单克隆抗体。在目的蛋白附近有一个接近的条带。想咨询一下，具体原因和可行的解决方案。谢谢

由E-mail来自我们的回复 Wednesday, Dec 23, 2020 - 13:28

老师您好

该抗体的结合位点大致在50-150位氨基酸之间，与N端剪切掉信号肽的Fibronectin type III domain-containing protein 5可以结合，且与进一步在C端发生剪切的Irisin也能结

PTM / Processing

Molecule processing

Feature key	Position(s)	Description
Signal peptide	1 – 31	Sequence analysis
Chain (PRO_0000328971)	32 – 212	Fibronectin type III domain-containing protein 5
Chain (PRO_0000415857)	32 – 143	Irisin

合。
此外，Isoform 2 中也包含50-150区段的氨基酸片段，因此该抗体也能识别Isoform 2

Isoform 1 (identifier: **Q8NAU1-1**) [UniParc] FASTA Add to basket

This isoform has been chosen as the canonical sequence. All positional information in this entry refers to it. This is also the sequence that appears in the downloadable versions of the entry.

« Hide

```
        10         20         30         40         50
MHPGSPSAWP PRARAALRLW LGCVCFALVQ ADSPSAPVNV TVRHLKANSA
        60         70         80         90        100
VVSWDVLEDE VVIGFAISQQ KKDVRMLRFI QEVNTTTRSC ALWDLEEDTE
       110        120        130        140        150
YIVHVQAISI QGQSPASEPV LFKTPREAEK MASKNKDEVT MKEMGRNQQL
       160        170        180        190        200
RTGEVLIIVV VLFMWAGVIA LFCRQYDIIK DNEPNNNKEK TKSASETSTP
       210
EHQGGGLLRS KI
```

Isoform 2 (identifier: **Q8NAU1-2**) [UniParc] FASTA Add to basket

The sequence of this isoform differs from the canonical sequence as follows:
180-181: KD → EA
182-212: Missing.

Show »

Chunye Chen
Scientific Support Specialist
www.abcam.cn
+86 21 20700500

We are committed to continually improving our service and value your input. You may receive a 'HaveYourSay' survey email. Please share your thoughts.

由E-mail来自您的咨询 Wednesday, Dec 23, 2020 - 10:05

2020-12-23

menxiaoming

发件人："Abcam Scientific Support" <cn.technical@abcam.com>
发送时间：2020-12-21 13:55
主题：我购买了ab174833，单克隆抗体。在目的蛋白附近有一个接近的条带。想咨询一下，具体原因和可行的解决方案。谢谢 [事件: 201220-000066]
收件人："menxiaoming"<menxiaoming@126.com>
抄送：

由E-mail来自您的咨询 Monday, Dec 21, 2020 - 13:55

您发给我的信件已经收到。

网易126邮箱--专业电子邮局，2280兆超大空间，支持超大附件，全国唯一24小时客户服务。
欢迎您来注册使用。网易126邮箱：http://www.126.com

This is an automatic reply, confirming that your e-mail was received.Thank you

由E-mail来自我们的回复 Monday, Dec 21, 2020 - 13:55

老师您好，

对于您使用ab174833于实验里没有获得理想结果, 我们感到非常抱歉。

根据UniProtKB - Q8NAU1 (FNDC5_HUMAN)（https://www.uniprot.org/uniprot/Q8NAU1#ptm_processing） 显示该蛋白存在20 kDa的isoform 2和剪切掉长为31个氨基酸的信号肽的片段，两者的大
掉信号肽片段的长度为32-212共181个氨基酸。若要进行实验验证需要选择其他免疫原的抗体，验证isoform 2 时需要使用免疫原在182-212之间的抗体，验证信号肽时需选择免疫原在1-31之间的抗体；此外若

我们查找到一篇文献在进行WB实验时也检测到多条带，您可查看其详细内容。PMID:29176631

希望以上回复能够帮助到您，您有任何问题可随时联系我们。

Chunye Chen
Scientific Support Specialist
www.abcam.cn
+86 21 20700500

We are committed to continually improving our service and value your input. You may receive a 'HaveYourSay' survey email. Please share your thoughts.

由E-mail来自您的咨询 Sunday, Dec 20, 2020 - 16:43

您发给我的信件已经收到。

网易126邮箱--专业电子邮局，2280兆超大空间，支持超大附件，全国唯一24小时客户服务。
欢迎您来注册使用。网易126邮箱：http://www.126.com

This is an automatic reply, confirming that your e-mail was received.Thank you

由自动答复 Sunday, Dec 20, 2020 - 16:42

感谢您联系Abcam! 我们已经收到您的咨询邮件，在多数情况下，您可望尽快得到答复。

您的查询编号为:201220-000066。如果您需要就此事再次联系我们，请参考这个参考号码，这将帮助我们查找到您最初的咨询。

为方便查询，您可在 http://www.abcam.com/account/inquiry 登入您的帐户，点击“查询 ”，便可看到过去六个月内您得到的

我们期待为您服务!

由E-mail来自您的咨询 Sunday, Dec 20, 2020 - 16:42

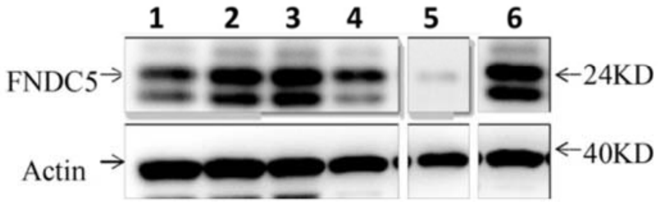

2020-12-20

nenxiaoming

Bai du 翻译 官网 抗疫行动 翻译 API 同传 上新啦 视频翻译 人工翻译 插件下载 APP下载 登录

检测到中文(简体) 英语 翻 译 人工翻译 通用

回复邮件1：根据UniProtKB - Q8NAU1 (FNDC5_HUMAN)（https://www.uniprot.org/uniprot/Q8NAU1#ptm_processing）显示该蛋白存在20 kDa的isoform 2和剪切掉长为31个氨基酸的信号肽的片段，两者的大小都约为20 kDa均可能导致其在WB中显现出双带。isoform 2的长度为1-181 共181个氨基酸，剪切掉信号肽片段的长度为32-212共181个氨基酸。

回复邮件2：该抗体的结合位点大致在50-150位氨基酸之间，与N端剪切掉信号肽的Fibronectin type III domain-containing protein 5可以结合，且与进一步在C端发生剪切的Irisin也能结合。此外，Isoform 2 中也包含50-150区段的氨基酸片段，因此该抗体也能识别Isoform 2。

回复邮件1：根据UniProtKB - Q8NAU1 (FNDC5_HUMAN)（https://www.uniprot.org/uniprot/Q8NAU1#ptm_processing）示该蛋白存在20 kDa的isoform 2和剪切掉长为31个氨基酸的信肽的片段，两者的大小都约为20 kDa均可能导致其在WB中显现双带。isoform 2的长度为1-181 共181个氨基酸，剪切掉信号肽段的长度为32-212共181个氨基酸。

Email 1: according to uniprotkb - q8nau1 (fndc5_ HUMAN)（https://www.uniprot.org/uniprot/Q8NAU1#ptm_ Processing）showed that isoform 2 of 20 kDa and signal peptide fragment o 31 amino acids were cut out, both of which were about 2 size, which may lead to the appearance of double band i The length of isoform 2 was 181 amino acids from 1 to 181, ar the length of signal peptide fragment was 181 amino acids fror 32 to 212.

意见反馈

回复邮件2：该抗体的结合位点大致在50-150位氨基酸之间，与端剪切掉信号肽的Fibronectin type III domain-containing protei可以结合，且与进一步在C端发生剪切的Irisin也能结合。此外，Isoform 2 中也包含50-150区段的氨基酸片段，因此该抗体也能别Isoform 2。

Email 2: the binding site of the antibody is about 50-150 aminc acids. It can bind to fibronectin type III domain containing prote 5, which cuts off the signal peptide at the N-terminal, and it car also bind to irisin, which further cuts off the signal peptide at th C-terminal. In addition, isoform 2 also contains 50-150 amino acids, so the antibody can recognize isoform 2.

报错 笔记 双语对照

排序 百度翻译邀您填问卷，领礼品！

重点词汇

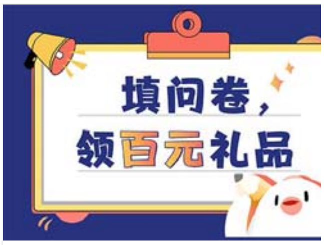

填问卷，赢百元礼品！

重点词汇

回复邮件	Reply All ; Reply to ; Reply with a message
剪切	shear ; shearing ; shear cut ; sheared
氨基酸	amino acid ; gamma-aminobutyric acid
信号肽	signal peptide
片段	part ; passage ; fragment ; extract
显现	manifest oneself ; reveal oneself ; appear ; show
回复	reply ; answer ; reflex ; reversion
邮件	mail ; post ; postal matter
大致	roughly ; on the whole ; approximately ; more or less
区段	sector ; zone

©2020 Baidu 使用百度前必读 | 百度wifi翻译机 | 百度大脑 | 百度智能云 | 翻译协议 | 建议与反馈 | 帮助
