# Supplementary material for: FNDC5 expression closely correlates with muscle fiber types in porcine longissimus dorsi muscle and regulates myosin heavy chains (MyHCs) mRNA expression in C2C12 cells
Source: PeerJ. 2021 Apr 19;9:e11065. doi: 10.7717/peerj.11065 (PMC8061570; doi:10.7717/peerj.11065)
Supplement: Supplemental Information 5 — The instructions of MycAway™ -Color One-Step Mycoplasma Detection Kit and detection results. [file peerj-09-11065-s005.pdf]

MycAway™ -Color One-Step Mycoplasma Detection Kit UNG Plus

一步法快速支原体检测试剂盒（防污染版）

产品信息

产品名称	产品编号	规格
MycAway™ -Color One-Step Mycoplasma Detection Kit UNG Plus	40612ES25	25 T
一步法快速支原体检测试剂盒（防污染版）	40612ES60	100 T

产品描述

MycAway™ -Color One-Step Mycoplasma Detection Kit UNG Plus（防气溶胶污染）是利用 Yeasen 独特的等温扩增技术，专门针对细胞培养液中支原体污染的快速检测产品。较以往支原体检测产品（货号：40611），本试剂盒中加入了 UDG 酶，以便去除含 dU 的 PCR 产物气溶胶污染，在极大程度上抑制假阳性反应，提高检测的准确度。而且加入的 UDG 酶在室温下起作用，且对温度敏感、易于灭活，不影响实验结果。

主要原理是若细胞培养物被支原体污染，支原体 DNA 的保守序列会被大量、快速地扩增，使反应液由蓝紫色变成天蓝色，结果肉眼可辨，无需电泳。

翊圣 MycAway™ -Color One-Step Mycoplasma Detection Kit UNG Plus（防气溶胶污染）可以检测多种支原体，包括细胞培养过程中常见的 8 种支原体。传统巢式 PCR 支原体法检测法易受细胞培养上清中抑制物的影响，产生假阴性结果；反应后需开盖电泳检测，增加污染导致的假阳性风险。一步法支原体检测试剂盒完全没有上述缺点，且其检测灵敏度、准确性远远高于 PCR 法。

产品组分

组分编号	组分名称	产品编号/规格	
		**40612ES25/25T**	**40612ES60/100T**
40611-A	MycAway™ -Color A	600 μL	600 μL×4
40611-B	MycAway™ -Color B	25 μL	25 μL×4
40611-C	Positive Control	10 μL	10 μL×4
40611-D	矿物油	500 μL	500 μL×4

运输与保存方法

冰袋运输。

-20 °C 避光保存，保质期 18 个月。若较长时间不用，请注意避光保存。

注意事项

1）使用本试剂前请仔细阅读说明书。

2）整个实验，应规范操作，包括反应体系的配制、样本处理及加样。

3）为了您的安全和健康，请穿实验服并戴一次性手套操作。

使用方法

1. 支原体待检样本准备

贴壁细胞：直接吸取上清。建议在细胞传代或换液 3 天以上，且汇合度达到 90%左右时取样，此时上清中支原体含量较高，便于检出。

悬浮细胞：500 g 离心 5 min 后，吸取上清。建议在细胞传代或换液 3 天以上时取样，此时支原体含量较高，便于检出。

2. 反应体系

将 MycAway^{TM} -Color 从-20 °C 取出，溶解后颠倒混匀，瞬时离心确保所有液体落到管底。根据待检测样品量，配制如下体系（通常实验需要设置阴性对照和阳性对照）：

	**单次反应体积（μL)**		**总体积（μL)**
**MycAway^{TM} -Color A**	24	× 样品数 × 1.1*	_____
**MycAway^{TM} -Color B**	1		_____

【*】：移液器存在误差，配制时请多出 10%为保证分装后每管的量。

3. 加样

待测样品：向其余反应管中加入 1 μL 待测培养上清；

阴性对照：第一支反应管中不加入任何样品，作为阴性对照；

阳性对照：向最后一个反应管中加入 1 μL Positive Control，作为阳性对照。

【注】：1) 如果反应是在水浴锅中进行，则每管加入 20 μL 矿物油，以防止液体蒸发导致结果的误差。如果反应是在 PCR 仪中进行，不需要加矿物油。

2）实验室中支原体污染十分常见，鉴于本试剂盒反应灵敏，建议用户在超净台中配制组分，以免出现假阳性结果。

4. 反应条件

加完样后，先将样品在 25~37 ℃放置 5 min，再在水浴锅或 PCR 仪中，恒温 63 °C 孵育 60 min。

【注】：本试剂盒所用酶对温度非常敏感，强烈建议使用 PCR 仪操作；如用水浴锅，应先加热使其达到规定温度后再反应。温差超过 2 °C，将导致扩增效率降低，使阳性反应无法在说明书规定的时间内达到典型的天蓝色。

5. 结果判断

63 °C 反应 60 min 后，立刻取出反应管，放于室温。在光线良好的环境中观察反应结果（建议以白纸为背景）。如果反应液仍为蓝紫色，则判定为阴性；若反应液为天蓝色，则判定为阳性。

【注】：在 63 °C 反应的时间必须准确计时，超过说明书规定的反应时间可能会出现假阳性。反应管不可开盖，否则会出现气空气中支原体污染反应，出现假阳性的现象。

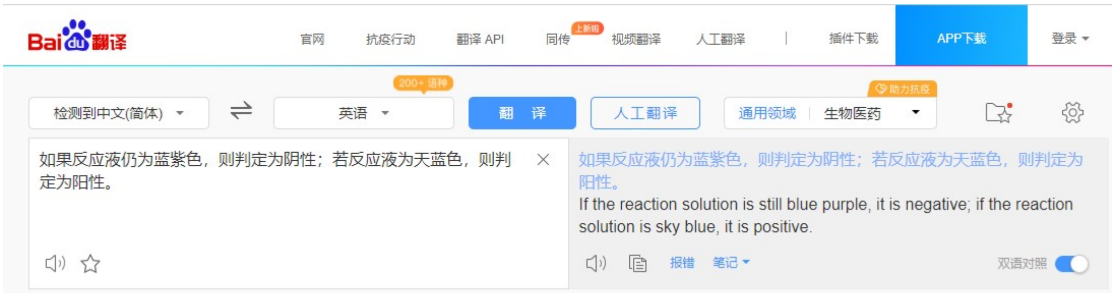

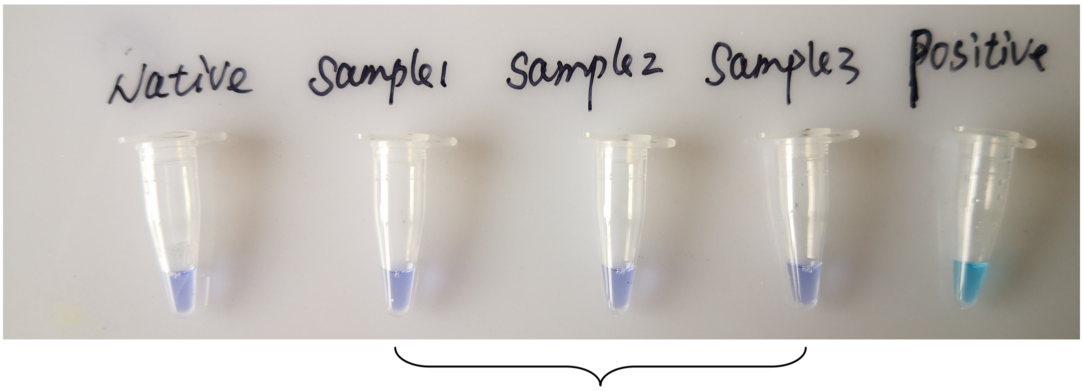

native: no mycoplasma
